# Treatment of AKI in developing and developed countries: An international survey of pediatric dialysis modalities

**DOI:** 10.1371/journal.pone.0178233

**Published:** 2017-05-30

**Authors:** Rupesh Raina, Abigail M. Chauvin, Timothy Bunchman, David Askenazi, Akash Deep, Michael J. Ensley, Vinod Krishnappa, Sidharth Kumar Sethi

**Affiliations:** 1 Department of Pediatric Nephrology, Akron Children’s Hospital, Akron, Ohio, United States of America; 2 Department of Nephrology, Cleveland Clinic Akron General, Akron, OH, United States of America; 3 Northeast Ohio Medical University, Rootstown, Ohio, United States; 4 Children’s Hospital of Richmond, VCU School of Medicine, Virginia, United States of America; 5 Department of Pediatrics, University of Alabama at Birmingham, Birmingham, Alabama, United States of America; 6 King’s College Hospital, Denmark Hill, London, United Kingdom; 7 Department of Political Science, Kent State University, Kent, OH, United States of America; 8 Kidney Institute, Medanta, The Medicity Hospital, Gurgaon, Haryana, India; University of Sao Paulo Medical School, BRAZIL

## Abstract

**Hypothesis:**

Acute kidney injury (AKI) is a common cause of morbidity and mortality worldwide, with a pediatric incidence ranging from 19.3% to 24.1%. Treatment of pediatric AKI is a source of debate in varying geographical regions. Currently CRRT is the treatment for pediatric AKI, but limitations due to cost and accessibility force use of adult equipment and other therapeutic options such as peritoneal dialysis (PD) and hemodialysis (HD). It was hypothesized that more cost-effective measures would likely be used in developing countries due to lesser resource availability.

**Methods:**

A 26-question internet-based survey was distributed to 650 pediatric Nephrologists. There was a response rate of 34.3% (223 responses). The survey was distributed via *pedneph* and *pcrrt* email servers, inquiring about demographics, technology, resources, pediatric-specific supplies, and preference in renal replacement therapy (RRT) in pediatric AKI. The main method of analysis was to compare responses about treatments between nephrologists in developed countries and nephrologists in developing countries using difference-of-proportions tests.

**Results:**

PD was available in all centers surveyed, while HD was available in 85.1% and 54.1% (p = 0.00), CRRT was available in 60% and 33.3% (p = 0.001), and SLED was available in 20% and 25% (p = 0.45) centers of developed and developing world respectively. In developing countries, 68.5% (p = 0.000) of physicians preferred PD to costlier therapies, while in developed countries it was found that physicians favored HD (72%, p = 0.00) or CRRT (24%, p = 0.041) in infants.

**Conclusions:**

Lack of availability of resources, trained physicians and funds often preclude standards of care in developing countries, and there is much development needed in terms of meeting higher global standards for treating pediatric AKI patients. PD remains the main modality of choice for treatment of AKI in infants in developing world.

## Introduction

Acute Kidney Injury (AKI) is common and imposes a heavy burden of morbidity and mortality. AKI diagnosis and treatment is complex due to varying definitions of the disease.

The worldwide incidence of pediatric AKI ranges from 19.3% to 24.1% [1], with mortality rates between 30% and 50% for AKI patients receiving renal replacement therapy (RRT) [2, 3]. Often, adult machines must be adapted to pediatric patients, and peritoneal dialysis is the only dialysis modality available [4]. Continuous renal replacement therapy (CRRT) is the mainstay treatment for severe pediatric AKI, but expense and complexity reduces accessibility; in a developing nation, one must often choose between available treatment and standard of care [5].

Three survey studies examining RRT modalities in pediatric patients compared three modes of pediatric dialysis: peritoneal dialysis (PD), hemodialysis (HD), and CRRT. These studies examined physician choice in RRT mode in North America, Europe and India [6–8], however there is a need for updated literature. Most literature currently available on RRT in children internationally consists of small, retrospective studies of non-random and non-generalizable populations. The purpose of this study was to conduct a survey to assess the trend of physician choice in mode of RRT use for AKI in children globally. We hope to provide more evidence surrounding global health in regards to RRT in pediatric populations, lending a modern perspective into the disparities and unique challenges developing countries face.

## Materials and methods

A team of pediatric nephrologists and intensivists from Asia, Europe, and the United States drafted an Internet based survey. The survey was piloted on 3 pediatric nephrologists and 2 pediatric intensivists at Cleveland Clinic Akron General. The survey utilized a convenience sampling method, and was sent via email link to the *pedneph* list serve (the world’s largest Pediatric Nephrology Online community), the *pcrrt* list serve (world’s only Pediatric online dialysis community), to local Pediatric Nephrologists in Asia via personal e-mail, and it was widely distributed during the pediatric nephrology meetings. The survey was self-conducted from October to December 2014, a 26-question “International Pediatric Dialysis Modality” survey was sent out to around 650 members internationally, of which 223 (34.3%) responded. Survey questions evaluated participants’ perspectives on availability, infrastructure and the common forms of RRT performed according to patient age and hemodynamic stability. Survey participation was completely voluntary, confidential, and non-compensated.

The survey first inquired about demographic information. Participants were asked about certification, and whether or not a pediatric nephrologist and RRT trained nursing staff were available at their location. Technological factors and availability of resources were evaluated as well: access to pediatric-sized technology and supplies, presence of a pediatric renal ward, modes of RRT available at their institution currently, and whether there were plans for future expansion. Additionally, clinical factors were analyzed, including cost of treatment and RRT modality choice in different categories of children. Demographic questions were short-answer, while the rest of the answer choices were presented in multiple-choice style for purposes of percentage distribution. Full survey is located in the appendix.

### Statistical analysis

The main method of analysis was to compare responses about treatments between nephrologists in developed countries and nephrologists in developing countries using difference-of-proportions tests. We report the *p* values associated with the hypothesis test that there is not a significant difference between the two proportions.

## Results

The summary of results is tabulated in Table 1 and Table 2. From a total of 223 responses (34.3% response rate), 60 came from U.S. centers and 48 from centers in India, Pakistan, Nepal, Africa and Latin America. The remaining responses came from the United Arab Emirates, Netherlands, Latvia, Auckland, Canada, China, Singapore, Thailand, Australia, Russia, Scotland, Serbia, Hungary, Italy, Finland, the United Kingdom, and Korea. Of the 48 responses from the developing world, 31 (64.6%) responses came from adult nephrologists who provide care for sick children in their area due to lack of pediatric nephrologist. Only 17 (35.4%) centers in the developing world had a trained pediatric nephrologist, compared to 100% (p = 0.00) of centers in the developed world. Dedicated pediatric HD units and trained staff were available in 16 of 48 (33.3%) developing world centers as compared to 159 of 175 (91%, p = 0.00) centers in developed world.

**Table 1:** Summary of surveys reporting pediatric dialysis modality use.

Author (year)	Goals	Response	Location	Modality Used
Belsha et al 1995 [7]	Describe pediatric AKI RRT	15 / 19 surveys returned	North America	PD– 31%
				HD– 25%
				HF– 44%
Warady et al 2000 [6]	Describe pediatric AKI management	92 / 123 surveys returned	North America and Europe	PD– 31%
				HD– 33%
				CRRT– 36%
Vasudevan et al 2012 [8]	Describe pediatric AKI RRT	23 / 26 surveys returned	India	PD– 83%
				HD– 17%
				CRRT/SLED– 5 to 10%

**Table 2:** Summary of results for RRT in AKI.

	Developing Countries	Developed Countries	p
**Availability of pediatric nephrologist**	35.4% (17/48)	100% (175/175)	0.000
**Availability of dedicated pediatric dialysis unit**	33.3% (16/48)	91% (159/175)	0.000
**Institute’s dialysis modality of choice in infants** PD	68.5% (33/48)	5.7% (10/175)	0.000
HD	12.5% (6/48)	72% (126/175)	0.000
CRRT	10.4% (5/48)	24% (42/175)	0.041
SLED	8.3% (4/48)	1.1% (2/175)	0.006
**Institute’s dialysis modality of choice in older children (>12 years old)** PD	29.1% (14/48)	22.2% (39/175)	0.319
HD	64.5% (31/48)	61.1% (107/175)	0.668
CRRT	2% (1/48)	14.8% (26/175)	0.016
SLED	2% (1/48)	2.2% (4/175)	0.933
**Availability of RRT’s** PD	100% (48/48)	100% (175/175)	1
HD	54.1% (26/48)	85.1% (149/175)	0.000
CRRT	33.3% (16/48)	60% (105/175)	0.001
SLED	25% (12/48)	20% (35/175)	0.452
**Indication for CRRT** Fluid overload in critically ill child	12.5% (2/16)	40% (42/105)	0.033
Hyperkalemia	81.2% (13/16)	100% (105/105)	0.000
Persistent metabolic acidosis	31.2% (5/16)	61.9% (65/105)	0.021
Hyperammonemia secondary to inborn errors and liver failure	100% (16/16)	100% (105/105)	1
**Prefered mode of CRRT** CVVH	12.5% (2/16)	17.1% (18/105)	0.637
CVVHD	43.7% (7/16)	14.2% (15/105)	0.004
CVVHDF	12.5% (2/16)	31.4% (33/105)	0.120
Depends on the clinical situation	25% (4/16)	35.2% (37/105)	0.422
**Change in dialysis modality choice in the past 10 years**	41.6% (20/48)	70.2% (123/175)	0.000
**Plans to add CRRT/HD services in the next 10 years**	10.4% (5/48)	0% (0/175)	0.000
**Access to newer dialysis machines (CARPEDIEM, Aquadex, NIDUS)**	0% (0/48)	2.28% (4/175)	0.291

The availability of RRT’s in the developing and developed world varied except for PD. PD was available in all the centers in both developing and developed world (100%) where as HD was available in 54.1% and 85.1% centers (p = 0.00); CRRT in 33.3% and 60% (p = 0.001) centers, and sustained low efficiency dialysis (SLED) in 25% and 20% centers (p = 0.45) in the developing and developed world respectively. Two-thirds of respondents (68.5%, p = 0.00) in the developing world report using PD as a first-line RRT for infants, while 96% of providers surveyed in the developed world use HD (p = 0.00) or CRRT (p = 0.04). The choice of PD and HD use in older children (>12 yrs) is higher in developing world than developed world, which is 93.6% and 83.3 respectively. This difference is due to more usage of CRRT in the developed countries (14.8%, P = 0.01) compared to developing countries.

The major indications for CRRT in both developing and developed countries are hyperkalemia (81.2% and 100%) and hyperammonemia (100% for both). Other less common indications are fluid overload (12.5% in developing and 40% in developed world) and persistent metabolic acidosis (31.2% in developing and 61.9% in developed world). Frequently used dialysate flow rates are 30 ml/kg/hr (81.2%) and 35 ml/kg/hr (43.7%) in developing world as apposed to ~2000–3000 ml/1.73 m2/hr (64.7%) and 35 ml/kg/hr (69.5%) in developed countries. Furthermore, developing world nephrologists report their choice of replacement fluid to be 80% pre-filter and 20% post-filter (62.5%) and 100% post-filter (31.2%) compared to developed world nephrologist who reported using 80% pre-filter and 20% post-filter majority of times (70.4%).

Most frequently opted mode of CRRT in developing countries is CVVHD (43.7%) followed by CVVH (12.5%) or CVVHDF (12.5%), however in developed world nephrologists most often prefer using CVVHDF (31.4%) followed by CVVH (17.1%) and CVVHD (14.2%). The most preferred anticoagulant of choice in both developing and developed countries is heparin (35.4% and 44% respectively, p = 0.28) and citrate is the second most preferred anticoagulant (29.7%, p = 0.007) in the developed world followed by normal saline. 97.1% of developed country respondents report using blood products to prime machines for CRRT compared to 12.5% of developing country respondents using blood to prime the circuit (p = 0.00).

20 centers (41.6%) in the developing world and 123 centers (70.2%) in the developed world report changes in modality preference over the last 10 years (p = 0.00), and 5 centers (10.4%) in the developing world report plans to add HD and CRRT machines in the next 10 years (p = 0.00). PD in infants in the developed world was achieved via a percutaneous soft catheter, while 40 of 48 centers in the developing world use acute rigid peritoneal dialysis catheters designed specifically for acute PD. Only 8 developing world centers use soft, Tenckhoff peritoneal dialysis catheter.

## Discussion

Comparative survey results of developing and developed countries are summarized in Table 2 and Table 3. Three survey studies about pediatric dilaytic modalities were reviewed (Table 1**)** [6–8] and compared with our survey results. In North America, Belsha, et al [7] reports that CRRT is preferred over HD and PD, however, our survey showed preference to HD in infants (72% of center, p = 0.00) and children >12 yrs old (61.1%, P = 0.66) in the developed world. This indicates that centers in other parts of developed world (except USA and Canada) still prefer HD despite availability of CRRT. Vasudevan, et al [8] reported that in India, even with the availability of PD, HD, CRRT, and SLED in 23 of the 26 centers surveyed, PD was the most commonly used modality due to lower cost and simplicity. This may also be due to lack of trained pediatric nephrologists. Further, McCulloch et al report PD as the primary RRT modality in Africa [9]. Findings from Vasudevan et al and McCulloch et al are similar to our findings that developing countries prefer PD in infants (68.5%, p = 0.15), however this trend is not observed in case of older children (>12yrs) where the modality of choice is HD (64.5%, p = 0.66). In contrast, Warady et al reported that although PD (45%) was preferred over CRRT (18%) for the treatment of pediatric patients with AKI in the past, survey results show a relatively even distribution of preference for PD (31%), HD (33%), and CRRT (36%), suggesting a growing trend in CRRT use and decreasing trend in intermittent HD (IHD) and PD usage [6]. Our survey partially supports Warady et al findings that CRRT is been used in 10.4% and 24% centers in developing and developed world respectively. In the same study, patient age was related to choice in mode of RRT: 64% of centers reported PD as their primary choice for children from 0 to 2 years and PD is the intervention of choice in >20% of centers for patients 12+ years of age [6]. Again, these finding are consistent with our survey results that 68.5% centers in developing world use PD as preferred modality of choice in infants, and in older children (>12 yrs), PD is used in 29.1% and 22.2% centers in developing and developed world respectively. All the three studies report that patient size, age, and resource availability strongly influenced modality choice [6–8].

**Table 3:** Dialysis parameters used for AKI.

	Developing Countries	Developed Countries	p
**Dialysate flow rates in CRRT**			
~2000–3000 ml/1.73 m2/hr	25% (4/16)	64.7% (68/105)	0.003
25 ml/kg/hr	12.5% (2/16)	10.4% (11/105)	0.800
30 ml/kg/hr	81.2% (13/16)	20% (21/105)	0.000
35 ml/kg/hr	43.7% (7/16)	69.5% (73/105)	0.042
**Blood priming in CRRT**	12.5% (2/16)	97.1% (102/105)	0.000
**Replacement fluid**			
80% pre-filter and 20% post-filter	62.5% (10/16)	70.4% (74/105)	0.523
50% pre-filter and 50% post-filter	12.5% (2/16)	10.4% (11/105)	0.800
100% post-filter	31.2% (5/16)	17.1% (18/105)	0.180
100% pre-filter	0% (0/16)	0% (0/105)	1
**Choice of anticoagulation**			
Citrate	10.4% (5/48)	29.7% (52/175)	0.007
Heparin	35.4% (17/48)	44% (77/175)	0.285
Normal saline pre filter	8.3% (4/48)	13.1% (23/175)	0.366
All	10.4% (5/48)	12% (21/175)	0.760

Most of the pediatric nephrologists in developed countries use blood to prime the circuit (97.1% Vs 12.5%, p = 0.00). Further, developed country pediatric nephrologists largely report using heparin (44%) as an anticoagulant in CRRT, despite evidence that citrate is a better regional anticoagulant [10, 11]. The strongest trend in CRRT was a lack of preference for continuous venovenous hemodiafiltration (CVVHD) or continuous venovenous hemofiltration (CVVH) in developed world.

## New dialysis technologies for infants and small children

To address the need of small extra corpuscular circuit (ECC) volumes imposed by small size of infants, 3 unique HD machines have been developed and their use has been tested; the Cardio-Renal Pediatric Dialysis Emergency Machine (CARPEDIEM), the Newcastle Infant Dialysis and Ultrafiltration System (NIDUS) and the Aquadex.

The CARPEDIEM, which is meant to replace PD in small children uses lesser priming volumes, more accurate scales and ultrafiltration, as well as small roller pumps: all of these components tailor the machine to the unique needs of such a small and young patient with renal insufficienc**y.** It has settings for three surface areas (0.075, 0.147, and 0.245 m^2^), and for circuit plus priming volumes (27.2, 33.5, and 41.5 ml) [12]. The CARPEDIEM is also unique in its capability to allow combination extracorporeal therapies such as blood or plasma exchange and single-pass albumin dialysis with continuous veno-venous hemofiltration or continuous veno-venous HD. Successful use of the CARPEDIEM has been reported in at least two newborn infants [13, 14]. Initial use was described in a 2.9 kg newborn with hemorrhagic shock, multi-organ failure and fluid overload secondary to vacuum extraction associated subgaleal bleed. Continuous veno-venous hemofiltration with CARPEDIEM was alternated with bilirubin-targeted therapies (blood exchange, plasma exchange and single-pass albumin dialysis for hepatic failure and hyperbilirubinemia) which lead to restoration of organ function 25 days later and subsequent discharge from hospital at 2 months of age with mild renal insufficiency [13]. Another 11-day-old newborn infant with multi-organ failure and severe metabolic acidosis due to septic shock was successfully treated with continuous veno-venous hemofiltration with the CARPEDIEM that lead to improvement after 5 days [15]. The presence of disseminated intravascular coagulation and gastrointestinal bleeding precluded using peritoneal dialysis in this patient.

NIDUS, first developed in 1995, uses two syringes to pump blood instead of a conventional peristaltic pump [16]. NIDUS contains automated smaller pump with a total ECC volume of <10ml without priming requirement. NIDUS is capable of decreasing ultrafiltration rate to microliter by repeatedly drawing 5 to 12.5 ml of blood across a high-flux polysulfone 0.045m^2^ filter, and the blood flow rate varies between 20–45 ml/min. Furthermore, NIDUS can be programmed to dialyzer operating pressure to reduce ultrafiltration rate in case of filter clotting. This auto feedback mechanism can also be programmed to immediately stop dialysis if air leak is detected in the circuit [17]. NIDUS, therefore, offers both precise and self-regulating dialysis using a single lumen access and requires less anticoagulation. Coulthard MG et al, in a randomized cross-over trial involving 7 piglets and 11 babies (weighing <8 kg), compared the efficacy of NIDUS with PD and conventional HD [17]. All the piglets were treated with both PD and NIDUS. Of the 11 infants, 6 had NIDUS treatment alone, 4 had both PD and NIDUS, and 1 infant required only PD. These 11 infants had total combined 192 hours of PD and 2,475 hrs of NIDUS during 354 treatment sessions. In both the piglets and infants, clearance of urea, creatinine and phosphate with NIDUS was consistently higher with minimal (microliter precision) variability compared to PD which was associated with wide variability. Conventional HD removed fluid imprecisely and was associated with hypovolemia needing correction.

The Aquadex HD machine was adapted and its efficacy in small children was reported by Askenazi et al [18]. The adapted Aquadex circuit has a small 33-ml volume when used with UF500 filter (surface area 0.12 m^2^) set. Pre-replacement fluids can be infused via the proximal pigtail of the circuit using an in-line medication infusion pump. Circuits were primed using saline or packed red cells diluted in a 1:1 ratio with bicarbonate. A total of 12 children with median age of 30 days and a median weight of 3.4 kg were treated with adapted Aquadex circuit. Seven of the 12 patients (58%) survived to come off of renal support or were switched to peritoneal dialysis. Of patients who survived, six lived to hospital discharge, while one died six months after treatment. None died as a consequence of renal failure or due to complication from use of adapted Aquadex circuit. Ashkenazi et al concluded that small ECC volumes involved during HD with adapted Aquadex circuit provided a safe ability to initiate therapy.

These new machines provide a superior and safe method of management in small and critically ill infants requiring HD. However, further research is needed to test efficacy and safety of these new technologies before their use in clinical practice routinely.

Our results showed that developing countries used PD as their RRT of choice in infants and at a larger frequency in older children when compared to PD use in developed countries. This could possibly be related to low income, lack of trained pediatric nephrologists and resource deficiency in developing countries, making PD the most effective and economical option [19]. In developed countries, HD is the predominant method of RRT across all age groups. Despite the disadvantages that developing countries face economically, steps are being taken to improve the standard of care in these locations. Through the efforts by the International Pediatric Nephrology Association (IPNA), Indian Society of Pediatric Nephrology, and International Society of Nephrology [9], as well as development of online resources and dialysis registries, the world is working to help to share information and encourage medical education [20].

## Conclusions

PD remains the primary modality of choice for treatment of AKI in infants in developing world and at a larger frequency in older children when compared to PD use in developed countries. Patients requiring RRT in developing countries are at a disadvantage due to lack of available resources, physicians, or funds.

## Limitation of the study

The study limitations are low response rate (34.3%) and lack of representation from major parts of Africa. The low response rate could be due to a variety of reasons, but in future studies further follow up could be of some benefit to obtain more responses.

## Supporting information

S1 File Survey questions. (DOCX)
