# Supplementary material for: Treatment of AKI in developing and developed countries: An international survey of pediatric dialysis modalities
Source: PLoS One. 2017 May 30;12(5):e0178233. doi: 10.1371/journal.pone.0178233 (PMC5448754; doi:10.1371/journal.pone.0178233)
Supplement: S1 File — (DOCX) [file pone.0178233.s001.docx]

**Survey questions**

1. Kindly tell about your current affiliation. The name shall be kept confidential, and shall not be shown in the results. The name is required just to avoid duplication of results, from multiple physicians from a single centre.

Institute name: ______________

State, Country: ______________

Your Name: ______________

1. Which of the following best describes your role as in the care of Pediatric Renal patients?
   1. Pediatrician
   2. Pediatric Nephrologist
   3. Adult Nephrologist
2. Do you have a trained pediatric nephrologist in your centre?
   1. Yes
   2. No, Managed by Adult Nephrologists
   3. No, Managed by Pediatricians
   4. No, We refer the patients to higher centres
3. Do you have trained nursing staff for pediatric renal patients and dialysis?
   1. Yes
   2. No
4. Do you have dedicated “renal ward” for pediatric renal patients?
   1. Yes
   2. No, but we are having plans to have dedicated pediatric renal beds in the future
   3. No, and we don’t have any plans to have dedicated pediatric renal beds in the future
5. Are the patients charged for the services provided to them for the renal replacement
   1. Yes, 100% are paid
   2. Yes, but they are at subsidised rate at our centre
   3. Not at all
   4. Yes, some beds are paid and some beds are free or subsidised based on the availability
6. Which of these renal replacement therapies are available in your institution? (Multiple options may be checked)
   1. Manual PD
   2. Automated PD
   3. Hemodialysis
   4. CRRT
7. What is your institute modality of choice in infants with acute renal failure?
   1. Hemodialysis
   2. Peritoneal Dialysis
   3. SLED
   4. CRRT
8. What is your institute modality of choice in older children with acute renal failure, who are hemodynamically stable?
   1. Hemodialysis
   2. Peritoneal Dialysis
   3. SLED
   4. CRRT
9. What is your institute dialysis modality of choice in pediatric renal failure with hemodynamic instability?
   1. Hemodialysis
   2. Peritoneal Dialysis
   3. SLED
   4. CRRT
10. Do you use automated peritoneal dialysis in your centre
    1. Yes, always when indicated in renal failure
    2. Sometimes, depends on availability of the machine
    3. Sometimes, depends on patient affordability to fluids and machine
    4. It is not available at our centre, but there are plans to buy automated machine in next 5 years
    5. It is not available at our centre, and there are no plans to buy automated machine in next 5 years
11. If your centre does not use Hemodialysis or CRRT as the initial modality, what is the reason for the non-use. You can choose and tick more than one option.
    1. We use Hemodialysis and CRRT both at our centre
    2. Lack of a dedicated pediatric dialysis unit
    3. Lack of trained manpower
    4. Patient choice
12. Has the choice of dialysis modality changed in your centre in the past 10 years?
    1. Yes, now we use more of Hemodialysis
    2. Yes, now we use more of Hemodialysis and CRRT
    3. No
13. If CRRT or HD not used in your centre, are there any plans to set up CRRT or HD in the next 10 years?
    1. Yes
    2. No
    3. Not sure
14. Attempt this question, if your centre does CRRT. Do you always blood prime in infants undergoing CRRT?
    1. Yes
    2. Sometimes
    3. No
15. Attempt this question, if your centre does CRRT. What is your preferred mode in CRRT?
    1. CVVH
    2. CVVHD
    3. CVVHDF
    4. Depends on the clinical situation
16. Attempt this question, if your centre does CRRT. What are your indications for initiation of CRRT? You can tick more than one response.
    1. Treating Fluid overload in a critically ill child
    2. Hyperkalemi
    3. Persistent metabolic acidosis
    4. Hyperammonemia in inborn errors and liver failure
    5. Prevention of fluid overload in a critically ill child
17. What Dialysate or effluent flow rates dose do you use in CRRT?
    1. ~2000-3000 ml/1.73 m2/hr
    2. 25 ml/kg/hr
    3. 30 ml/kg/hr
    4. 35 ml/kg/hr
18. What anti-coagulation do you use? (Multiple options may be checked)
    1. Citrate
    2. Heparin
    3. Normal saline pre filter
    4. All
19. In case of using replacement fluid as an effluent how do you divide replacement fluid?
    1. 80% pre-filter and 20% post-filter
    2. 50% pre-filter and 50% post-filter
    3. 100% post-filter
    4. 100% pre-filter
20. What is an average filter life while patient on CRRT with anticoagulation?
    1. Less than 12 hrs
    2. 12-18 hrs
    3. 24-48 hrs
    4. 48-72 hrs
21. What modality do you chose in setting of septic patient with MODS?
    1. CVVH-D
    2. CVVH
    3. CVVH-DF
    4. Anyone
22. Do you calculate Filtration Fraction – The UF rate/plasma flow rate [=BFRx(1-HCT)] ratio and keep at < 0.35-0.4 in order to avoid filter clotting
    1. Yes
    2. No
23. Do you use SLED or SLED-F
    1. No
    2. Only SLED
    3. SLED-F
    4. Both
24. Do you have smaller hemodialysis tubes available in your country for infants and children?
    1. Yes
    2. No
25. Do you have access to these Pediatric specific filters/machines?
    1. HF20
    2. AV Peds Frensenius
    3. CARPEDIEM
    4. Aquadex
    5. NIDUS
